# Valorization of Biomass-Derived Polymers to Functional Biochar Materials for Supercapacitor Applications via Pyrolysis: Advances and Perspectives

**DOI:** 10.3390/polym15122741

**Published:** 2023-06-19

**Authors:** Caiyun Yang, Hao Wu, Mengyu Cai, Yuting Zhou, Chunyu Guo, Ying Han, Lu Zhang

**Affiliations:** 1 Hebei Key Laboratory of Heavy Metal Deep-Remediation in Water and Resource Reuse, Hebei Key Laboratory of Applied Chemistry, School of Environmental and Chemical Engineering, Yanshan University, Qinhuangdao 066004, China; 2 Jintong Internet of Things (Suzhou) Co., Ltd., Suzhou 215000, China

**Keywords:** biochar, biomass-derived polymers, electric double layer, supercapacitor, surface modification

## Abstract

Polymers from biomass waste including plant/forest waste, biological industrial process waste, municipal solid waste, algae, and livestock are potential sources for renewable and sustainable resources. Converting biomass-derived polymers to functional biochar materials via pyrolysis is a mature and promising approach as these products can be widely utilized in many areas such as carbon sequestration, power production, environmental remediation, and energy storage. With abundant sources, low cost, and special features, the biochar derived from biological polymeric substances exhibits great potential to be an alternative electrode material of high-performance supercapacitors. To extend this scope of application, synthesis of high-quality biochar will be a key issue. This work systematically reviews the char formation mechanisms and technologies from polymeric substances in biomass waste and introduces energy storage mechanisms of supercapacitors to provide overall insight into the biological polymer-based char material for electrochemical energy storage. Aiming to enhance the capacitance of biochar-derived supercapacitor, recent progress in biochar modification approaches including surface activation, doping, and recombination is also summarized. This review can provide guidance for valorizing biomass waste to functional biochar materials for supercapacitor to meet future needs.

## 1. Introduction

**Figure 1:**
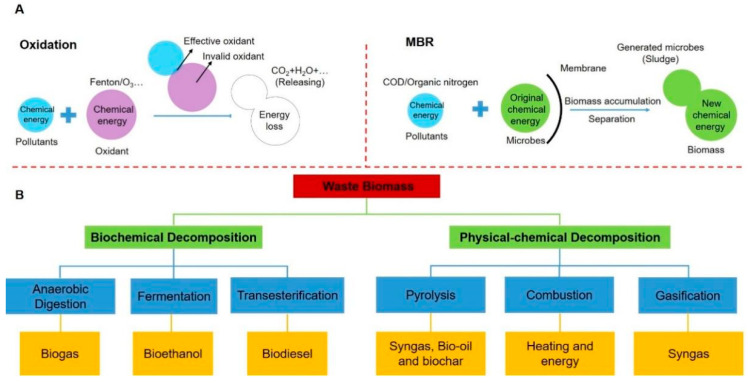
(**A**) Energy loss in conventional pollution removal methods, such as advanced oxidation and MBR. (**B**) Steps involved in conversion of bioenergy and valuable products from waste biomass.

In recent years, the rapid population expansion and industrial/economic development have caused a series of severe problems in environment and energy [1,2]. The backward waste management mode and the increasing energy demand from societies and industries have led to a higher consumption of fossil fuels over pass decades [3]. Notwithstanding the advantages such as easy accessibility, compatibility, and affordability of fossil fuels, they are nonrenewable sources, may be exhausted in the future, and cause excessive greenhouse gas emissions [4,5]. Therefore, converting waste to green and efficient products, as well as developing renewable and environmentally friendly technologies for energy generation, storage, and utilization are global essential issues that need to be faced. However, conventional waste treatment methods only focus on removing or destroying the pollutant molecules, whereas the chemical energy contained in pollutants is ignored and wasted during the pollutant’s degradation process [6,7]. The wasted energy may present as extra power consumption, high cost in the physical–chemical process, or extra biomass generation during the biological process (Figure 1A), which violates the initiative of carbon neutrality. Therefore, it is critical to find suitable technologies to satisfy the increasingly diverse, global waste treatment, and sustainable energy demand.

Currently, many waste materials are utilized as alternative feedstocks for producing chemicals, materials, and energy, among which polymeric material derived from waste biomass is considered to be a source for clean energy and substitute for valuable product [8,9,10]. The sources of waste biomass polymers involve residues from plants, forest waste and production, process industries, the organic fraction of municipal solid waste, and the algae and livestock sector [11]. Approximately 181 billion tons of biomass waste is produced annually globally, presenting the dual property of being both resources and pollutants, as the energy in polymeric components of biomass is the most abundant source of renewable energy (14–18% renewables in energy mix), accounting for 10% of the total global energy supply [12]. However, the abundant, easily degradable matter in biomass and its inevitable generation may cause serious environmental and sanitation issues [13]. Therefore, converting the polymeric components in biomass to valuable products is of great importance in both waste management and resource recycling.

The current refinery approaches for polymers and organics in biomass waste mainly consist of biochemical processes and thermochemical processes (Figure 1B). Polymeric components in waste biomass can be fermented to produce bioethanol and biodiesel or digested to generate biogas via biological processes [14,15,16]. They can also be used for heat generation to produce bio-oil, syngas, and biochar via thermochemical processes such as pyrolysis, gasification, and hydrothermal carbonization [8,17]. As a typical product from the thermochemical treatment of biomass polymeric components, biochar has attracted global attention due to its advantages of sustainability, abundant sources, cost-effectiveness, excellent physical–chemical stabilities, and specific morphology, and it is widely applied in many carbon-demanding areas including CO_2_ capture, carbon sequester, soil remediation and amendment, wastewater/waste gas treatment, function material synthesis, climate change mitigation, and energy storage [8,12,17,18,19]. Its typical applications are summarized in Table 1. Although the property of biochar is controlled by the particular polymeric structure of raw biomass, it can be easily tuned by changing the thermochemical treating condition, gifting it various modification possibilities [17].

Currently, to promote the beneficial use of various renewable energy sources for power generation, the development of electrochemical energy storage systems that can efficiently handle electricity storage and output has become essential. A supercapacitor (SC) is a typical representative of these novel energy storage systems widely used in smart consumer electronics and vehicles, which has attracted global attention due to its advantages of high power density, long cycle life (>30,000 h), and quick charge/discharge capacity (1–10 s) [29,30,31]. Unlike conventional capacitors and renewable batteries, the SC is important in bridging the gap between battery and conventional capacitor, and it is particularly suitable for power systems that require high power throughput but relatively low energy density as it can achieve much higher energy or power density than a conventional capacitor or battery (shown in Table 2). The performance of an SC is highly controlled by the property/quality of its electrode material and electrolyte, and research on carbon materials with high performance such as graphene, carbon nanotube, and carbon quantum dot has attracted more attention as an SC electrode [32,33,34]. However, such kinds of high-quality carbon materials are usually difficult to prepare, and the cost may be too high for mass production. Thus, seeking an alternative electrode material with low cost and high performance is necessary.

Polymeric components in biomass waste are promising precursors for high-quality biochar-based electrode material preparation. Postprocessing modifications are essential for the resultant biochar material to meet the performance requirements of SC. Therefore, this review systematically summarizes the preparation and characteristic of biochar using polymeric components in biomass waste, as well as essential strategies for polymeric structure postprocessing modification to fill the gap between different types of SCs and biological polymer-derived biochar materials. In addition, this review provides an overview of recent studies regarding biological polymer-derived biochar-derived SCs especially for flexible SCs with self-healing functions, as well as further perspectives to promote the development of biological polymers for energy storage.

## 2. Biochar Production from Polymers in Waste Biomass

### 2.1. Technologies of Biomass Polymers Derived Biochar Preparation

Biochar is a solid product obtained from the thermochemical conversion of biomass in an oxygen-limited environment in the case of aerobic carbon oxidation [29]. The available polymeric components in biomass for biochar preparation are complex and vary from their different sources, with polysaccharide, hemicellulose, cellulose, and lignite as their main constitutions [1]. Technologies that can thermochemically convert these polymeric substance to biochar involve pyrolysis (slow and fast), gasification, hydrothermal carbonization, etc. [35].

Pyrolysis is a dry process to decompose biomass thermally under oxygen-free conditions (N_2_, Ar, CO_2_, or air with O_2_ consumed) at a temperature range of at least 300 °C [36]. During the pyrolysis process, each polymeric component of biomass (i.e., cellulose, hemicellulose, and lignin) undergoes its own pathway at a defined temperature, resulting in the generation of biochar, bio-oil, and mixed syngas. The biochar yield dramatically depends on the adaption of the pyrolysis type (Table 3). Slow pyrolysis performed in a wide temperature range (300–800 °C) with a relative long residence time (>1 h) and low heating rate (5–7 °C/min) results in higher biochar yield [37]. Fast pyrolysis is the thermal decomposition of biomass at medium temperatures with a high heating rate (>300 °C/min) and short residence time, resulting in higher bio-oil [18]. Gasification is a thermochemical partial oxidation process converting the polymers in biomass to gaseous products using gasification agents (including oxygen, air, steam, or mixtures of these gases) under high temperature. Biomass gasification is an effective method to produce syngas; thus, the biochar yield is usually very low (less than 10% of the total biomass) [11]. Unlike dry carbonation of biomass polymers, hydrothermal carbonization uses subcritical water (temperature varied from 100 to 374 °C) to carbonize the polymeric components of biomass in a closed vessel with pressures ranging from saturated steam to 22 MPa to maintain water in liquid form. The products depend on the operation temperature, and biochar is the main product at around 250 °C [4]. Furthermore, technologies of flash carbonization and torrefaction are also widely applicated in biomass polymeric component carbonization [38].

Since the formation of biochar significantly depends on the feedstocks and reaction conditions in the thermochemical treatment of biomass polymers, a reasonable and economic design for biomass polymeric components conversion process is essential. The biochar mass yield, energy recovery rate, and fixed carbon content can be used to evaluate the production and energy recovery effectiveness, as well as biochar quality, during a biomass conversion process [39]. The calculation of mass yield (y_m_) uses the following equations:

(1) $$y_{m}=\frac{m_{\mathrm{bioc}}}{m_{\mathrm{biom}}}\times 100\%,$$

(2) $$y_{m}=\frac{w_{\mathrm{ash},\mathrm{biom}}}{w_{\mathrm{ash},\mathrm{bioc}}}\times 100\%,$$

(3) $$y_{m}=\left(1-\frac{1-\frac{w_{\mathrm{ash},\mathrm{biom}}}{w_{\mathrm{ash},\mathrm{bioc}}}}{1-w_{\mathrm{ash},\mathrm{biom}}}\right)\times 100\%,$$

where m represents the mass of raw biomass or biochar, and w stands for the ash content in raw biomass or biochar.

Equation (1) is used for general biochar yield calculation; however, if the biomass contains high contents of water, the yield calculated from Equation (1) will be very low. Thus, if the yield is not directly measured by gravimetric analysis, it can be calculated by the ash content of raw biomass and biochar product, as shown in Equation (2). Equation (3) provides a yield calculation based on the ash-free part of biomass and shows how much organic matter in the raw biomass remains as solid biochar.

Usually, the biochar preparation process is cost-effective and environmentally sustainable because energy consumption in pyrolysis can be compensated for through the generation of other types of bioenergy. The energy yield (y_e_) denotes how much energy in the feedstock remains in the biochar product, and it can be calculated using the mass yield and the heating values of feedstock and product, as shown in Equation (4).

(4) $$y_{e}=y_{m}\times \frac{{\mathrm{LHV}}_{\mathrm{bioc}}}{{\mathrm{LHV}}_{\mathrm{biom}}},$$

where LHV is the lower heating value of raw biomass or biochar product.

In addition, the quality of biochar can be evaluated via its carbon content as it totally comes from the feedstock. Thus, the fixed carbon yield relates to the carbon content in biochar and the total organic carbon in feedstock [8], which can be expressed as follows:

(5) $$y_{c}=y_{m}\times \frac{w_{c,\mathrm{bioc}}}{w_{c,\mathrm{biom}}}.$$

### 2.2. The Formation Mechanism of Biomass Polymer Biochar

The overall mechanism for biochar formation is the combination of pyrolysis for each polymeric component in biomass. The temperatures and conversion pathways of the representative polymeric components and elements in biomass during the biochar formation process are summarized in Table 4. The results indicate that the pyrolysis of cellulose and hemicellulose is first, whereas the pyrolysis of lignin is complex and involves free-radical reactions [18,40]. Some special biomasses such as manure and sewage sludge contain no noteworthy amounts of such polymeric components due to their different origins; hence, they need to be characterized and treated differently.

### 2.3. Effect of Pyrolysis Conditions on Biochar Formation

**Figure 2:**
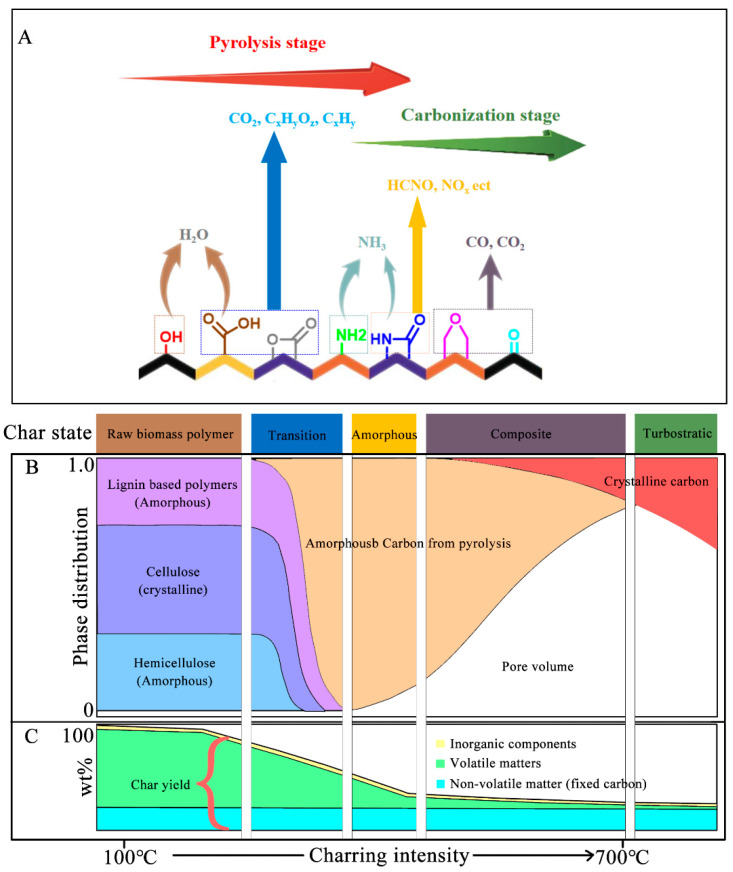
Dynamic change in the functional groups and composition of the biochar produced in different thermal conditions: (**A**) decomposition of biomass with increasing pyrolysis temperature; (**B**) changes in physical and chemical composition (include components, phases, and crystal morphology); (**C**) changes in biochar composition as inferred from gravimetric analysis.

In addition to the polymeric biomass components, the pyrolysis conditions also crucially affect the characteristics of biochar products. Pyrolysis temperature is a primary factor that controls the formation and characteristic of biochar. As shown in Figure 2A, pyrolysis treatment induces the emission of volatile matters (moisture, inorganic gases, VOCs, etc.) and the evolution of functional groups to remove the impurities from biomass. Therefore, biochar product tends to produce low yield but high carbon content under intense pyrolysis treatment [8]. Weber and Quicker [39] summarized the carbon content variation of biochar product made from polymers in woody and straw-like biomass under different pyrolysis temperatures (from 200 °C to 700 °C). The result indicated that both the carbon contents of the two biochar products increase with pyrolysis temperature; the carbon contents increased from less than ~50% to ~90% for woody and from ~48% to ~88% for straw-like biomass with increasing pyrolysis temperature.

Pyrolysis conditions also control the crystal structure of biochar. Carbon materials such as graphite and graphene exhibit high electrical conductivity, and their conductive mechanism is based on the movement of delocalized π electrons in a large-scale conjugation system within a planar crystal structure [8]. Usually, the biochar structure mainly consists of a large amount of amorphous structure (randomly organized aromatic rings) and some conjugated aromatic crystalline structure (condensed poly-aromatic sheet) [41]. Different biochars have differing degrees of aromatic linked sheet development (graphitic sheets) and irregularities (comprised of layer, carbon isomerism, substitutions and edge defects) in the rhombohedral crystalline network [42]. As shown in Figure 2B, intense charring will increase the content of highly conjugated aromatic crystalline in biochar to arrange the entire structure toward being more ordered [18]. Thus, the delocalization electrons can move between aromatic rings localized on distinct neighboring planes, which is like graphite, giving biochar a better conducive property. Chen et al. [43] prepared three kinds of biochar (namely BEC, ESI, and Klin) using pine as a feedstock with different pyrolysis temperature conditions, and the thermal condition for BEC preparation was 700 °C for 30 s then 500 °C for 15 min, that for ESI was 500 °C for 2 h, and that for Klin was 600 °C for 2 h. The results suggested that biochar with higher pyrolysis exhibits higher electrical conductivity; thus, the electric conductivity for BEC, ESI, and Klin was 4.41, 2.11 and 4.33 μS/cm, respectively. In addition to the improvement of biochar electrical conductivity by intense charring treatment, the increase in the biochar’s aromaticity and aromatic condensation content will enhance its environmental stability. High contents of energetically stable aromatic ring structures may extend or cluster into a large poly-condensed unit, which is crucial for biochar stability in the environment [17].

However, an intense charring process may cause a decline in biochar surface properties. Increasing pyrolysis temperature is helpful to enlarge the surface area of biochar, but intense treatment will condense the structure of biochar, thereby decreasing surface area. Brown et al. [44] analyzed the characteristics of biochar produced in different thermal treating processes. The results indicated that biochar produced at low temperature exhibited poor surface area (<10 m^2^/g), while the surface area gradually increased with pyrolysis temperature. Biochar produced at around 600–750 °C had a maximum surface area of around 400 m^2^/g. A further increase in temperature could lead to a decrease in surface area (details mainly depend on the original feedstock and thermal condition), and the surface area of biochar produced at over 1000 °C was lower than that produced at low temperature. In addition to surface area, the change in electrical conductivity was analyzed in their study; the resistivity of biochar product decreased by five orders of magnitude between 600 and 750 °C, and by around seven orders of magnitude at 1000 °C. The possible explanation is indicated in Figure 2B,C, whereby some pore structures can form when pyrolysis removes the volatile matter from polymeric feedstocks, which increases the surface area at low temperature. The pyrolysis process causes deformation that results in a widening of the micropore by destroying the walls between adjacent pores, which increases the surface area and total pore volumes under high temperature. An intense charring process promotes the conversion of crystal from amorphous carbon into ordered turbostratic crystallites to guarantee a high current density of the SC by increasing the electrical conductivity, as well as a dense carbon structure, causing a decline in biochar surface area [8].

### 2.4. Element Composition of Biomass Polymeric Component-Derived Biochar

The main elements of biochar are C, H, and O (sometimes including a minor N component) [17,18]. The exact content of C, H, and O greatly depends on the raw components in biomass polymer; the typical element composition of biochar contains 45–60 wt.% C, 10–20 wt.% O, and 2–5 wt.% H [45]. The element compositions of biochar are indicative of the type of C–C bonds, aromatic C content, and functional groups; thus, it is important for evaluating the stability and further utilization potential of biochar. Total carbon is a general property of biochar due to the thermal treatment of polymers in biomass favoring dehydration and deoxygenation. Thus, the result of biomass polymer pyrolysis is to eliminate H and O and to accumulate C in the final product. The International Biochar Initiative adopts an upper H/C limit of 0.7, which can guarantee biochar with abundant fused aromatic ring structures, and distinguish it from the raw polymeric feedstock or other partially deficiently carbonized materials [46]. Yang et al. [8] suggested that the H/C and O/C ratios can be used to calculate the unsaturation or aromaticity degree of biochar, and the ratio values correlated negatively with the percentage of aromatic C in biochar. Oxygen is also an important element in biochar as it significantly affects the stability, structure, and surface physical–chemical properties of biochar. For example, graphite exhibits high stability and electric conductivity due to a low O/C ratio of almost 0 (totally non-detected or <0.5 wt.%); however, for biomass polymer-derived biochar, it is usually around 0.6 or higher [17]. In addition, the structure of biochar materials is relatively complex as they are not graphite-like and contain some relic structures from the biomass; therefore, the actual biochar is not a single entity but has large variability of compositions in terms of both structure and chemical composition. Biochar with a lower O/C ratio has higher stability and ordered turbostratic crystallites; for instance, biochar with an O/C ratio lower than 0.2 can possess an estimated half-life of more than thousands of years, whereas a ratio higher than 0.6 results in the half-life of biochar usually being shorter than 100 years (easily degraded) [42].

The N, S, and P content in biomass polymeric components also influences the biochar formation and function. The initial biomass contains many organic compounds with O- or N-based functional groups including carboxylic acids, phenols, alcohols, aldehydes, ketones, amides, amines, and heterocycles. These O- and N-based functional groups increase the surface reactivity and correspondingly decrease the stability of biochar. S- and P-containing functional groups would react, volatize, and condense during the biomass thermal treatment, producing liquid bio-oil and biochar with rich aromatic C structures. Moreover, H, N, O, P, and S are incorporated into these aromatic structures, influencing the surface chemical electronegativity of the biochar product that, in turn, affects the surface charge of biochar [18,29]. In addition to the elements mentioned above, some inorganics elements are present in biochar (Si, Al, Fe, Ca, Mg, K, Mn, etc.), which more or less affect the formation and properties of biochar [8].

## 3. Postprocessing Modification of Biomass Polymer-Derived Biochar for Energy Storage

As discussed above, the characteristics of biochar mainly depend on the raw feedstock of biomass polymeric components and the thermal treatment conditions; however, biochar products just from simple pyrolysis usually exhibit a poor surface functionality, with limited porosity or surface area (usually <150 m^2^/g) [45]. These disadvantages limit the application potential of biochar as a functional material, especially as the electrode material of SCs. Thus, postprocessing modification is necessary to valorize raw biochar to a high-value material for SCs, and the modification strategies are based on the energy storage mechanism of different types of SCs.

**Figure 3:**
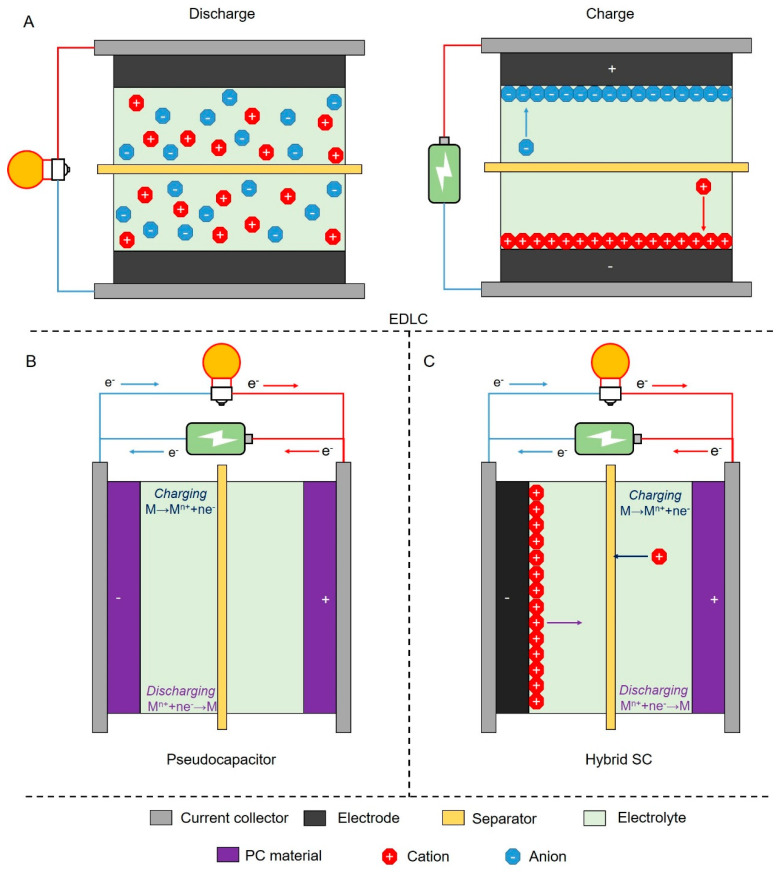
Schematic illustration of the working mechanisms of SCs: discharging/charging process in (**A**) EDLC, (**B**) PC, and (**C**) hybrid supercapacitor.

The electric double-layer capacitor (EDLC), pseudo-capacitor (PC), and hybrid capacitor (Figure 3) are typical SCs with different working mechanisms. An EDLC, which typically employs carbon-based materials, is based on ion adsorption/desorption at the electrode–electrolyte interface that leads to fast charge/discharge and a stable cycle life [47]. A PC is produced by a Faradaic charge storage mechanism, presented in a series of fast and highly reversible surface or near-surface redox reactions that result in increased power/energy density compared with EDLCs [48]. The Faradaic reaction often occurs in electrochemically active materials such as metal oxides and conducting polymers. A hybrid capacitor comprises two electrodes that electrostatically and Faradically store charges, thus combining the advantages of the previous two mechanisms [49].

According to the working mechanism, the performance of SC significantly depends on the properties of electrode material and electrolyte. Fortunately, the surface functionality and property are easily tuned via a series of strategies, which makes biochar a promising platform for the further preparation of various useful materials. Studies regarding biochar postprocessing modification for SC application usually focused on the activation and surface functionalization of biochar to improve surface area, pore structure, and surface function.

### 3.1. Biochar Activation

The activation process controls the specific surface area and pore distribution for biochar material to meet the requirement of energy application. It is reported that the mobility of ions into pores is greatly affected by the pore size of biochar material, and not all pores are accessible to ions [50]. Some studies suggested that, if the pore size on the electrode surface is <0.5 nm, hydrate ions do not have access [51]. In addition, a pore size smaller than 1 nm is not accessible for most organic electrolytes [52]. Arguments also exist from this viewpoint; for instance, Chmiola et al. [53] claimed that the capacitance can be greatly increased with pore size below 1 nm. This viewpoint explains that the increase was due to a reduction in the distance between charges by the distortion of the solvation shell [54]. Moreover, it is widely accepted that a well-defined pore size distribution (mesopore or micropore wider than 2 nm, or 2–50 nm) can enhance the power capability of an SC for a rapid supply of electrolyte to the pore surface where the main charge storage occurs [55]. Thus, to improve the surface area and adjust the pore structure, activation is the most used method. Activation is the selective gasification of carbon atoms of biochar, removing the low-molecular-weight carbon molecules and, thus, generating voids in the material structure. The methods of biochar activation can be divided into (1) physical activation and (2) chemical activation [56].

Physical activation can provide biochar with a moderate to high porosity and varying surface chemical properties. It utilizes oxidizing gases (including O_2_, air, steam, CO_2_, and O_3_) to partially gasify the biochar products with a temperature range of 350–1000 °C [57,58,59]. The mechanism of physical activation is to promote the development of pore structure and the burning of active sties on the biochar surface via controlled oxidation [56]. Commonly, a high activation temperature and long activation time can enhance the surface area and pore structure development [29]. Braghiroli et al. [57] used CO_2_ as an active agent to prepare activated and nonactivated biochar with birch and spruce wood in a pilot-scale experiment; the results indicated that the surface area for birch and spruce biochar increased from 177 to 881 m^2^/g, and from 208 to 896 m^2^/g, respectively. Zhu et al. [58] used softwood sawdust polymers to prepare biochar in an atmosphere of N_2_, and the as-synthesized biochar was then activated with air at 600–800 °C. The results indicated that activation at 700 °C with an air flux of 50–80 mL/min was favorable for mesopore development; the BET surface area increased from 389 to 586 m^2^/g, while the microporous surface area was enhanced from 316 to 462 m^2^/g. The final mesoporous surface area and pore volume were 316 m^2^/g and 0.284 cm^3^/g, respectively.

The chemical activation process involves mixing the as-formed biochar with a chemical activating agent, followed by a thermal process at around 400–900 °C [29,56]. The advantages of chemical activation are the high surface area (up to 3600 m^2^/g) and developed microporosity with a narrow distribution. The chemical activating agents are usually common chemicals such as acids, bases, and salts (including H_3_PO_4_, KOH, K_2_CO_3_, and ZnCl_2_) [60,61,62]. KOH, H_3_PO_4_, and ZnCl_2_ are the most used among these various chemical activating agents. KOH is a well-known activating agent in biochar postprocessing modification, and it usually acts as an oxidant to generate the pore structure in carbon materials. The activated mechanism was well reviewed in a published article by Wang and Kaskel [63], and it involved the penetration of metallic K into carbon lattices, the expansion of lattices by the intercalated metallic K, and the removal of the intercalated K from the carbon matrix. Unlike KOH, the role of H_3_PO_4_ and ZnCl_2_ in the activation process is as a dehydration agent. H_3_PO_4_ or ZnCl_2_ promotes dehydration in a range of temperature lower than the thermal treatment, leading to biochar structure reduction; subsequently, the remaining activation agents in the carbon structure act as a template to generate the pore structure. In conclusion, activation with KOH, H_3_PO_4_, and ZnCl_2_ leads to unique characteristics; activation with KOH only widens the microporosity to more heterogeneous micropores, whereas activation with ZnCl_2_ develops both wide micropores and small mesopores, and activation with H_3_PO_4_ develops large mesopores and even macropores [56]. Luo et al. [64] compared the activation effect of KOH, H_3_PO_4_, and H_2_O_2_ on a pinewood polymer-based char with a total surface area of 232.2 m^2^/g, a total pore volume of 0.138 cm^3^/g, a microporous surface area of 129.9 m^2^/g, and a microporous volume of 0.07 cm^3^/g. Their result indicated that the three activation agents obviously increased the surface properties of biochar, and the most significant improvement in pore structure was obtained by KOH treatment, yielding a total surface area of 1124.4 m^2^/g, a total pore volume of 0.723 cm^3^/g, a microporous surface area of 923.6 m^2^/g, and a microporous volume of 0.485 cm^3^/g.

Moreover, physical activation and chemical activation can be combined. In the first step, the use of chemical agents allows the generation of narrow micropores without obviously changing the density of biochar, and the subsequent physical activation step appropriately promotes the development of the primary pores generated from the first step. Arami-Niya et al. [65] reported the preparation of high-performance activated charcoal from the polymeric components of oil palm shell using a combined chemical–physical activation method. H_3_PO_4_ and ZnCl_2_ were used as the activation agents for chemical activation, followed by a physical activation process with CO_2_. The results indicated that, compared with a single activation process using CO_2_, the combined activation process was more favorable for increasing surface area and promoting the development of a microporous structure.

### 3.2. Biochar Modification

#### 3.2.1. Surface Doping

Surface modification is a promising method that can not only change the surface characteristic but also give biochar materials a special function. Biochar surface doping incorporates hetero atoms into the bulk lattice of biochar to introduce specific functionality for specific applications. Similar to the activation process, biochar can be doped with oxygen atoms on its surface by oxidation to increase the number of oxygenated functional groups such as carbonyl, hydroxyl, carboxyl, lactones, and peroxide. During this process, H_2_O_2_, O_3_, KMnO_4_, and nitric acid are commonly used surface oxidation reagents [18,66]. The doped oxygen atoms in functional groups can interact with several ions via hydrogen bonding or complexation to improve the adsorption of biochar. Moreover, such groups can enhance the hydrophilicity of biochar material, thus improving its performance in liquid electrolyte for SCs [67]. In addition to surface oxidation, biochar surface doping includes surface amination and sulfonation. For instance, doping N atom into the carbon structure of biochar can significantly increase the stability and performance of heavy metal adsorption, CO_2_ capture, catalysis, and energy storage [12]. S-doped biochar is a promising starting material to prepare solid acid. The densities of the acid sites in biochar-based material can reach 2.5 mmol H^+^/g; thus, biochar-based solid acid has the potential to be used as a solid-state electrolyte for an all-solid-state SC. Phosphorus is also an alternative element for biochar surface doping. Compared with N and S, the atomic radius of P (0.110 nm) is larger, which results in a large layer spacing when P is doped into the carbon of biochar. P–O groups may form a thin layer on the carbon surface. Moreover, P atoms usually exhibit an *sp^3^* configuration, resulting in a twisted and open edge morphology which can provide more active sites for electrochemical and storage properties [68]. In addition to O, N, S, and P, some studies have explored doping chloride (Cl) and fluorine (F) onto biochar material. Cl can inhibit the agglomeration of partially graphitized biochar and increase the accessibility of electrolyte electrodes. However, when the atomic ratio of Cl is high, the desorption of electrolyte ions will be reduced. Doping F can improve the conductivity of biochar by greatly reducing the charge transfer resistance because F can induce the redistribution of some heteroatoms as a function of its high electronegativity [68,69].

#### 3.2.2. Surface Recombination

Surface recombination with metal oxides/hydroxides or loaded nanoparticles is another strategy for biochar surface modification. Some high-valent metals preloaded on biomass polymers can be reduced to zero-valent metals or low-valent metal oxides/hydroxides by organic components in feedstocks during biochar formation. Thus, their reduction products are loaded in situ on the surface of biochar, yielding a biochar with surface recombination [70]. Metal oxides/hydroxides or nanoparticles have been reported to show great potential in SC application; the variable oxidation states of metals help in charge storage through electrochemical reactions.

Transition metal oxides and hydroxides based SCs usually follow the PC mechanism, while carbon materials usually follow the EDL mechanism [71]. When loading metal or metal oxides/hydroxides on carbon material, the SC possesses both pseudo-capacitance and EDL capacitance. Loading functional nanoparticles on biochar surface can increase the surface redox activity, along with reducing the agglomeration of the nanoparticles [29]. In addition to transition metal-based nanoparticles, some nanostructures can be loaded on the surface of the biochar by linking with the functional groups via chemical reactions [72], which may improve the surface properties and redox performance of biomass polymer-derived biochar material.

## 4. Recent Advances in Biochar-Based SCs

The EDLC and hybrid SC are representative biochar-based SCs characterized by different energy storage mechanisms. The capacitance of an EDLC depends on the electric conductivity, specific surface area, pore structure, and chemical stability of biochar. The capacitance of PC is mainly attributed to the contact surface area and redox reactions between electrode and electrolyte [73,74]. Thus, this section reviews the development of recent carbonized biomass polymer-derived SCs.

### 4.1. Biochar-Derived EDLC

Studies regarding to biomass-derived EDLC often focused on the surface modification of raw biochar as the performance of EDLC significantly depends on its electrode material. Qiu et al. [75] synthesized a porous carbon-based EDLC electrode from corn straw polymers by flash pyrolysis. After 1 h of chemical activation with KOH at 800 °C, the optimized resultant porous carbon exhibited a BET surface area of 2790.4 m^2^/g, a microporous surface area of 568.4 m^2^/g, a mesoporous surface area of 2221.6 m^2^/g, a total pore volume of 2.04 cm^3^/g, a microporous volume of 0.08 cm^3^/g, a mesoporous volume of 1.96 cm^3^/g, and a conductivity of 23 mS/cm. The prepared SC exhibited a maximum specific capacitance of 327 F/g for 12,000 cycles. Qu et al. [59] reported an EDLC with an electrode made from waste corncob residue; the as-formed biochar was further activated in steam. The final SC electrode had a high surface area of 1210 m^2^/g and exhibited a high capacitance of 314 F/g in 6 M KOH electrolyte. Moreover, the SC showed almost no capacitance decay after 100,000 cycles.

Some studies used natural or artificial additive as a template to synthesize crosslinked polymeric biochar material for SCs. Gao et al. [76] used crab shell as a feedstock to prepare crosslinked biochar. The natural CaCO_3_ component in crab shell acted as an in-situ template to fabricate mesoporous biochar with high surface area. The CaCO_3_ template was decomposed after pyrolysis and removed by acid/water washing to leave a high-porosity crosslinked C structure. The biochar material possessed a high surface area of 634 m^2^/g and a mesoporous percentage of 70.80%. The maximum capacitance of the crab shell biochar-based SC reached 220 F/g. Liu et al. [77] prepared a cotton-based biochar polymeric fiber using the macrocosmic structure of cellulose as a template. The difference is that they used NaOH/urea solution to pretreat the cotton biomass in situ to improve the surface characteristics. The NaOH/urea treatment caused the swelling of cotton tissues to modify the degree of natural crystallinity and dimensions of unique crystallites without changing the crystal structure of cellulose, which increased the internal surface area of the biochar product by decreasing the degree of cellulose crystallinity and polymerization. The swollen biochar product showed improved surface parameters compared to the non-swollen group, and the surface area, microporous area, total pore volume, and pore diameter were 584.99 m^2^/g, 436.8 m^2^/g, 0.3846 m^3^/g, and 2.63 nm, respectively. These parameters in the control group were only 136.66 m2/g, 9.561 m^2^/g, 0.1607 cm^3^/g, and 4.70 nm, respectively. The SC derived from the swollen biochar exhibited an enhanced capacitance of 221.7 F/g at 0.3 A/g, and only 4.6% loss was observed after 6000 cycles at 2 A/g.

In addition, in the work of Liu et al. [77], the biochar they prepared possessed an interconnected hollow hierarchical porous structure which could enable a high ion-transport capability across the electrodes. Unlike conventional porous materials that cannot be easily adjusted under a relatively narrow range of length scales, hierarchical porous materials have the advantage of consisting of pores with different sizes, whose structures are based on various length scales and/or all kinds of morphologies. The interconnected porous structure similar to an ion reservoir can provide low-resistance pathways for ion transportation at high rates [78]. Moreover, the large space of the porous structure can enhance the electrostatic adsorption area [50]. For example, Bai et al. [79] used sodium alginate/bacterial composite polymeric cellulose as a feedstock and KOH as an activation agent to produce hierarchical porous biochar for SC. The surface characteristics increased upon increasing the operation temperature from 700 to 900 °C, and a hierarchical porous structure was obtained with a BET surface area of 1870 m^2^/g, a microporous area of 654 m^2^/g, a total porous volume of 2.03 cm^3^/g, and a microporous volume of 0.2 cm^3^/g. The corresponding capacitance was 259 F/g at 0.5 A/g.

**Figure 4:**
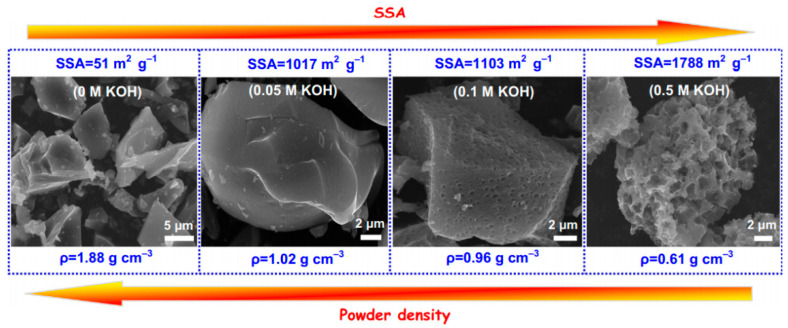
The trend of specific surface area (SSA) and bulk density of biochar material. An increase in surface area usually drives a decrease in bulk density. Reprinted from Long et al. [80]. Copyright 2015 Elsevier.

SCs with smaller volume and lighter weight, but higher power output are always in demand around the world. Although biochar electrodes with high surface area and abundant porous volume perform high EDL capacitance by enhancing the ion storage capacity, carbon materials with high surface area and porous volume possess a relatively low mass density (less than 0.5 g/m^3^) (Figure 4). Therefore, the capacitance of an EDLC is usually high in terms of gravimetric performance, but low in terms of volumetric performance. In addition, the low volumetric power/energy density may result in a space-limited electrolyte or an oversized device. Considering only the gravimetric power performance is insufficient to evaluate the performance and the potential for the practical use of a SC, and it is important to evaluate the SC performance using a volumetric scale. Developing biochar with a densely porous structure provides a potential option for overcoming this issue. For instance, Long et al. [80] reported the conversion of an abundant, eukaryotic fungus (*Auricularia*) to a densely layer-stacked carbon nanosheet (<10 nm stacking layer thickness) with an interconnected porous network via hydrothermal treatment. In addition to serving as an activation agent, KOH was introduced into fungus cells to act as in-built template that prevented adjacent cell-wall fusion or agglomeration. This fungal polymer-derived biochar exhibited a high bulk density of around 0.96 g/cm^3^, a high surface area of 1103 m^2^/g, and an ultrahigh volumetric capacitance of 360 F/cm^3^.

### 4.2. Biochar-Derived Hybrid SCs

In addition to EDLC, the combination system of EDLC/PC derived from biomass polymer is a hot topic that has attracted the attention of many researchers. Originally, the concept of PC only existed in SCs with an electrode made from PC material including metal oxides and conducting polymers [71]. The capacitance of PC is severalfold higher than that of EDLC; however, the poor cycle life and mechanical stability are challenges when using a Faradaic PC. Synergistically integrating PC material with biochar by dispersedly coating PC material on the surface of biochar porous structure can significantly offset the density loss caused by the large porous volume in biochar and enhance the performance/cycle life by decreasing the agglomeration of functional PC material particles.

**Figure 5:**
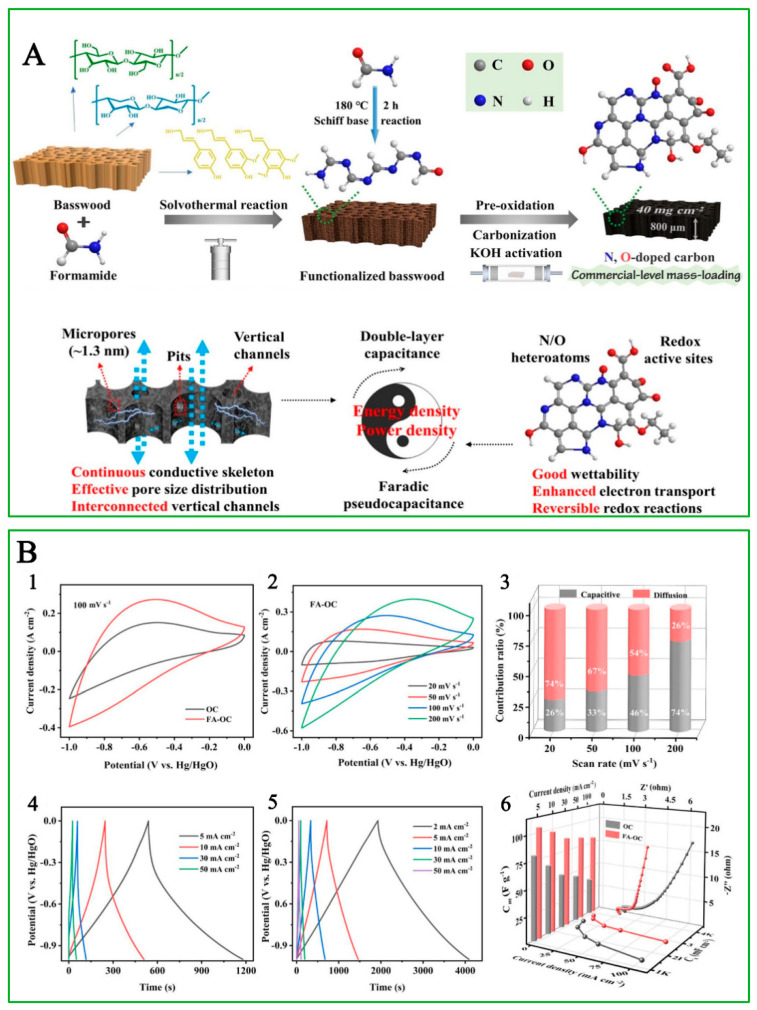
(**A**) The design concept and fabrication process of N-doped O-rich basswood-derived SC. (**B**) The comparison of CV curves and capacitive contribution of FA-OC and OC (1, 2, and 3), the GCD profiles of OC and FA-OC (4 and 5), and the rate performance and the EIS plots of OC and FA-OC (6). Revised and reprinted from Yan et al. [81].

A typical phenomenon of Faradic pseudo-capacitance is generated from biochar materials with surface doping. To offset the density loss in porous biochar material and develop effective electrodes with commercial-level active mass loading (>10 mg/cm^2^), Yan et al. [81] prepared a self-standing biochar electrode with 800 μm thick basswood. The basswood was treated by formamide to incorporate N and O into the carbon structure, followed by medium KOH activation to ameliorate the pore size and dope more O atoms into the carbon matrix. The resultant biochar monoliths (material named FA-OC) possessed a highly conductive carbon skeleton, abundant N/O atom dopant, and a 3D porous structure, which were favorable for the ion/electron transportation (Figure 5A). Compared with non-formamide-treated material (OC), the cyclic voltammetry (CV) curve of FA-OC exhibited a larger enclosed area at a fixed scan rate of 100 mV/s, which indicated a more obvious pseudo-capacitance donated by N/O dopant (Figure 5(B1,B2)). When increasing the scan rate, the CV window areas exhibited an increasing trend, and the capacitive/diffusion-controlled contributions indicated that the pseudo-capacitive contribution of FA-OC electrodes increased to 74% at 20 mV/s, and it was retained at 26% at 200 mV/s (Figure 5(B3)). In addition, as the scan rate increased, the CV curves were gradually deformed due to the slow redox kinetics of heteroatom-containing functional groups, and the solvated electrolyte ions did not have enough time to desolvate and enter the ultra-micropores. The galvanostatic charge/discharge (GCD) profiles (Figure 5(B4,B5)) at different current density indicated that the increased capacitance of FA-OC at low current density was due to the combined effects of ion desolvation in ultra-micropores and greater O doping ratio, along with additional N doping, while the improved capacitance retention at high current density benefited from the improvement effect of N–Q and N–X species on the conductivity of the carbon matrix. The rate performance and the electrochemical impedance spectroscopy (EIS) profiles indicated that the R_s_ of FA-OC and OC was nearly 1 Ω, indicating a small internal resistance assigned by high-temperature pyrolysis (Figure 5(B6)). In the high-frequency region, the R_ct_ related to the charge transfer resistance of FA-OC was smaller than that of OC. In the low-frequency region, the slope of the fitted EIS curves of FA-OC was steeper than for OC, indicating their smaller diffusion resistance, higher electrical conductivity, and faster ion diffusion rate, which were responsible for the improved rate capability. The assembled SC exhibited a maximum areal/mass/volumetric specific capacitance of 5037.5 mF/cm^2^, 172.5 F/g, and 63.0 F/cm^3^ at 2 mA (0.05 A/g), respectively [81]. Doping with electron-donating or electron-withdrawing atoms in C structure could also change the electrical properties of biochar material. For example, oxygen functionalities are often present on the surface of biochar; they are hydrophilic and can participate in the interactions between electrode and electrolyte ions, thus increasing the capacitance of biochar, especially in an acidic aqueous environment [8,73]. In the work of Bai et al. [79], as mentioned previously, although the highest surface characteristic was obtained at 900 °C, biochar sample with a thermal treatment of 700 °C exhibited the highest capacitance (302 F/g) due to the highest content of O-containing functional groups. N-containing surface functional groups in biochar can enhance capacitance in both aqueous and organic electrolyte. The doped elements can also come from the raw biomass feedstock. For example, Lin et al. [82] used magnesium acetate (C_6_H_6_MgO_4_) as a multifunctional template for one-step conversion of onionskin to hollow mesoporous biochar nanocages for application as PC electrodes. The N, O, and S contained in onionskin was self-doped on the resultant nanocages to improve the wettability, conductivity, and pseudo-capacitance effect of the SC electrode. The C_6_H_6_MgO_4_ activation conferred the resultant biochar an interconnected porous structure with a 2D multilayer wall/3D hollow nanocage featuring abundant active heteroatoms and cage defects. The specific structure exhibited a high BET surface area of 1369 m^2^/g and a total porous volume of 1.81 cm^3^/g. The electrical analysis implied that the pseudo-capacitance from N, O, and S broke the electroneutrality of neighbored C atoms and created many faradic active sites. As a result, the capacitance was 478.2 F/g at 0.5 A/g.

Metal oxides/hydroxides are also common PC materials. Nirmaladevi et al. [83] prepared biochar-supported MnO_2_ nanorod composites using wood biomass and KMnO_4_ during an in situ synthesis method. The thin film from this composite showed a high specific capacitance of 512 F/g, an energy density of 74.3 Wh/kg, and a power density of 2 kW/kg. This high electrochemical performance was attributed to the high surface area from the biochar matrix and the increased redox active sites provided by MnO_2_ nanorods. In addition to oxides/hydroxides, polyoxometalate can be used to modify porous biochar for preparing PC electrodes. Genovese and Lian [84] prepared KOH-activated porous biochar using pinecone as a feedstock; then, the porous structure was tuned by adsorbing phosphomolybdic acid solution, and a PMo_12_O_40_^3−^-loaded porous biochar electrode was synthesized. An ultrahigh content of PMo_12_O_40_^3−^ (55 wt.%) in the biochar matrix imparted tremendous redox activity to the already large EDL biochar substrate, leading to a high area capacitance of 1.19 F/cm^2^ for the hybrid material. The ratio of pseudocapacitive and Faradic contributions of the composite in the optimized condition was 4.5, and the gravimetric capacitance was 361 F/g.

Another type of PC material is conductive polymer. The electric conductivity of conductive polymer comes from a highly conjugated bond matrix in its structure. Like metal oxides/hydroxides, although conductive polymers can provide high pseudo-capacitance, their shortcoming is a low cyclic stability during the charge or discharge process. Furthermore, they are limited by the poor surface area of bulk conductive polymers, and the direct utilization of conductive polymers is ineffective for PC [85]. These challenges can be minimized by combining conductive polymers with biochar to form a composite structure. Zhao et al. [86] used dandelion fluffs as precursors to prepare activated carbon tube bundles with a porous hollow structure. Then, polyaniline (PANI) was used to modify the activated carbon tube bundles via an in-situ method. The PANI on the surface of the carbon material was beneficial to enhance its available area for redox reaction, leading to a high pseudo-capacitance. Moreover, the highly retained hollow porous tube structure promoted the diffusion rate of ions in electrolyte. When tested as an asymmetric SC, the composite displayed a high specific capacitance of 592 F/g (at 1 A/g) and a high energy density of 42 Wh/kg with high capacitance retention after 10,000 cycles. In addition to PANI, polypyrrole (PPy) can be used in biomass-derived SCs. The authors of [87] reported a PPy-anchored carbon aerogel and its application in high-performance SC. The cattail pretreated with NaClO_2_/HAc was used as a feedstock to prepare biomass-derived carbon aerogelsl then, PPy was loaded on the carbon aerogel by in situ oxidative polymerization. The PPy–biochar composite displayed the maximum areal capacitance of 419 mF/cm^2^ in 1 M H_2_SO_4_ electrolyte with excellent cycling. Some representative cases regarding biochar-derived SCs are shown in Table 5.

### 4.3. Biochar-Derived Flexible and Self-Healing SCs

**Figure 6:**
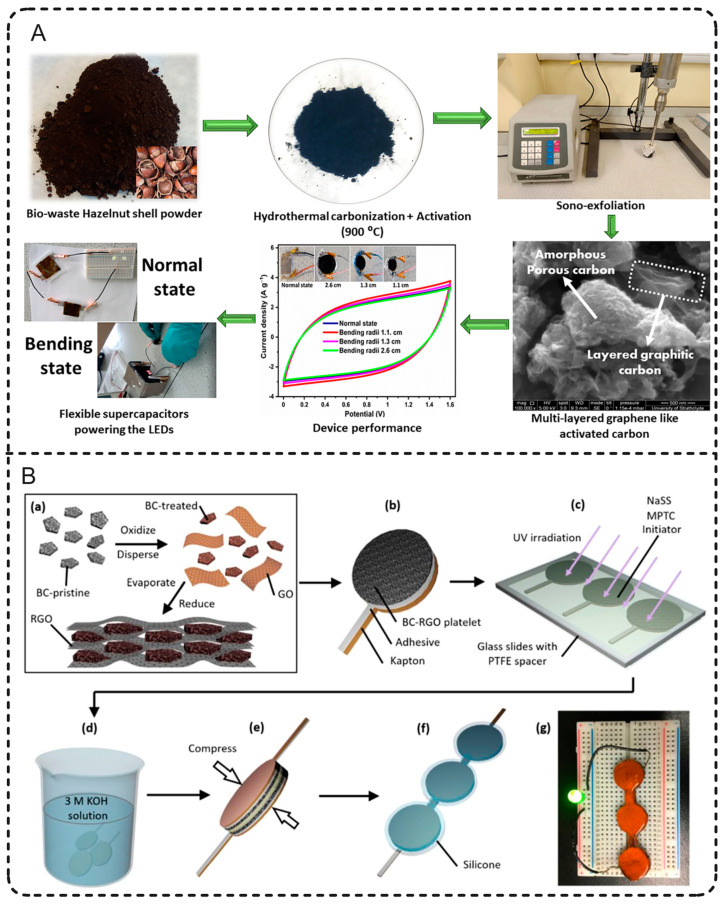
Fabrication schematic of biomass-derived flexible SCs. (**A**) A flexible hybrid SC and its performance in plate and bending state. (**B**) SC with self-healing function. Reprinted from Reddygunta et al. [98] and Li et al. [102].

Currently, flexible and portable electronics have shown broad application prospects; thus, the demand for energy storage devices with flexible and portable characteristics has grown stronger and stronger [98]. Conventional SC design, especially for a liquid-state electrolyte, seriously limits biochar-derived SCs in flexible and portable applications. For example, SCs may become invalid when inorganic electrolyte solution leaks or dries up. Some organic electrolytes or ion liquids used in low-temperature conditions are flammable, toxic, and expensive [99]. Thus, there is a critical need to improve both electrode material and electrolyte to meet the requirements of flexible SCs. Fabricating SCs with a flexible electrode and solid-state electrolyte is a typical solution. For example, Reddygunta et al. [98] fabricated an all-solid-state flexible supercapacitor using KHCO_3_-pretreated hazelnut hydrochar electrode, hydroxyethyl cellulose, and Na_2_SO_4_-containing biopolymer electrolyte (Figure 6A). Mechanical exfoliation led to the biochar exhibiting a graphene-like structure with a high surface area. The supercapacitor exhibited a dual performance of EDLC and PC, attributed to the porous carbon matrix and N atoms in the raw biomass polymer, respectively. Its capacitance was 320.9 F/g at 0.2 A/g of current density with an energy density of 38.7 W h/kg at a power density of 198.4 W/kg.

Another issue for the practical application of most SCs is that the electrode material will break due to bending or charge/discharge processes, which will lead to performance deterioration and a safety risk [100]. Fabricating SCs with self-healing material can not only prolong the service life of SCs but also improve the reliability of a product and reduce the waste of resources [101]. Li et al. [102] used biochar-based composite electrode and a polyampholyte hydrogel electrolyte to fabricate a flexible and self-healing SC. As shown in Figure 6B, treated biochar was dispersed in a graphene oxide solution; subsequently, a consolidated electrode with high electric conductivity was prepared by evaporating the solvent and reducing graphene oxide. The as-prepared electrode was further loaded on a Kapton tape. Then, a polyampholyte hydrogel was synthesized as the electrolyte on the surface of electrode and dialyzed in KOH solution. After compressing the dialyzed electrolyte/electrode pair, a flexible SC was made. The flexible SC showed a high energy density of 30 Wh/kg with around 90% capacitance retention after 5000 cycles. At room temperature, the power density was 50 W/kg, and the specific capacitance was 216 F/g. The polyampholyte hydrogel electrolyte had preferable mechanical properties such as stretchability, tear resistance, and adjustable adhesion, and it could self-heal when snapped.

## 5. Conclusions and Perspectives

Converting the biopolymers in waste biomass to biochar is attractive for both environmental and energy efficiency reasons. As the main constituent element is carbon, biochar is expected to be an alternative material for expensive activated carbon, graphene, and carbon nanotube in the electrodes of SCs. In this review, the synthesis, postprocessing modification, and application of biomass polymer-derived biochar in SC were concisely summarized. To satisfy the energy demand in modern technology and meet the requirement of high power density, fast charge/discharge speed, and long cycle life for SCs, a good management of biomass polymer valorization is necessary and essential. The following items are highly recommended:

(1) Stability and electric conductivity are important properties for biochar as an electrode material that affect the power output and cycling life of SC. Biomass polymer carbonization at a relatively high thermal treatment temperature and long retention time is necessary to rearrange the skeleton of polymers and generate a large area, high aromatic content, and conjugated, ordered crystalline structure in biochar.

(2) It is important to further develop the surface area and porous structure of biochar via physical/chemical activation as the surface characteristics of raw biochar materials are usually limited, significantly affecting the EDL capacitance of biochar. A large specific surface area and suitable porous size (e.g., <1 nm, or from 2 nm to 50 nm) will lead to maximum EDL capacitance.

(3) Volumetric power performance is equally important to gravimetric power performance. To avoid the bulk density decrease caused by high surface area and porosity, a decoration of the tuned surface area and porous structure by introducing PC material is significant. The decoration can be achieved by doping inorganic heteroatoms on the surface or C structure (which can be inferred during the biochar production process), or by loading metal oxide/hydroxide nanoparticles/structures and conductive polymers on the biochar surface. The decorated biochar electrode will display a dual capacitance with a long cycle life, and an increase in volumetric power performance.

The further development of SCs may involve increasing power output (both power and energy density), lowering the weight, reducing the size, and enabling potential applications in extreme environments with lower cost. These challenges should be overcome without losing the high cycle life and exceptional rate performance of SCs. Therefore, extensive work regarding biochar material optimization, decoration with functional groups and nanoparticles, and development of new electrodes and electrolytes need to be conducted in the future. In addition, valorizing the polymers derived from actual waste biomass for SC will facilitate benefits in terms of both environment and energy.

## Figures and Tables

**Table 1:** A summary of the potential applications of biochar material.

Application	Purpose	Reference
**Catalyst**		
Syngas purification	Removing syngas tars	[20]
Liquid biofuel production	Converting syngas to liquid hydrocarbons	[21]
Biodiesel production	Preparing solid acid catalyst for biodiesel production	[22]
**Soil amendment**		
Mitigating GHG emissions	Sequestering solid carbon to mitigate GHG emissions	[19]
Increasing soil quality	Increasing soil fertility, pH of acidic soil, and soil cation exchange capacity, and improving soil microbial activity and nutrient retention	[23]
**Adsorbent**		
Pollutant removal	Absorbing contaminates in soil, water, and gases	[8]
**Gas adsorbents**		
Storage material	CO_2_ sequestration	[24]
	H_2_ storage	[25]
**Fuel cell system**		
DCFC *	Used instead of fossil fuel for power generation	[26]
Microbial fuel cell	Enabling a carbon-negative circular economy and lowering the electrode cost	[27]
**Raw material**		
Activated carbon	Producing AC with low cost	[28]
**Energy storage**		
Supercapacitor	For long-life, quickly charging/discharging power supply	[12]

* DCFC, direct carbon fuel cell.

**Table 2:** Comparison of typical capacitor, SC, and battery characteristics.

Characteristic	Capacitor	SC	Battery
Specific energy (W h/kg)	<0.1	1–1091	10–1606
Specific power (W/kg)	>>10,000	500–19,600	<1000
Charging time	10^−3^–10^−6^ s	s to min	0.3–3 h
Discharging time	10^−3^–10^−6^ s	s to min	1–5 h
Coulombic efficiency (%)	Around 100	85–99	70–85
Cycle-life (cycles)	Infinite	>500,000	Around 1000
V_max_ determinants	Dielectric thickness and strength	Electrode and electrolyte stability window	Thermodynamics of phase reactions
Charge stored determinants	Electrode area and dielectric	Electrode microstructure and electrolyte	Active mass and thermodynamics

**Table 3:** Comparison of different biochar production processes.

Process	Temperature (°C)	Residence Time	Biochar Yield (wt.%)
Slow pyrolysis	300–800	min to days	20–40
Fast pyrolysis	400–600	Around 1 s	10–20
Gasification	800–1000	5–20 s	<10
HTC *	180–250	1–12 h	30–60
Flash carbonization	300–600	<30 min	~40
Torrefaction	Around 290	10–60 min	61–84

* HTC means hydrothermal carbonization.

**Table 4:** Pyrolysis pathways of the main components and elements in biomass.

Components	Temperature (°C)	Pathway
Cellulose	200–260	Cellulose → oligosaccharides → d-glucopyranose → levoglucosan → levoglucosenone → biochar
Hemicellulose	240–350	Hemicellulose → oligosaccharides → 1,4-anhydro-d-xylopyranose → biochar
Lignin	280–500	Lignin → vanillin/2-methoxy-4-methylphenol → biochar (via β-O-4 linin linkage-based radical reaction) *
K and Cl	Low	Vaporization
Ca and Mg	High	Ionically or covalently bounding with organic compound, or vaporization
P, S, and N	Low	Decomposed

* As the observation of radicals in pyrolysis is difficult, to clarify the exact mechanism in this process remains a challenging task.

**Table 5:** Studies on biochar material postprocessing modification and the performance of biochar-derived SCs.

Electrode Material	Modification	Capacitance * (Scan Rate)	Electrolyte (Conc.)	Energy Density Power Density * at (Current Density)	Stability after (Cycle Num.)	Ref.
*Osmanthus* flower	KOH	255 F/g (5 mV/s)	KOH (6 M)	7.95 Wh/kg 10 kW/kg (20 A/g)	92.9% (10,000)	[88]
Litchi seed	CO_2_ activation	493 F/g (10 mV/s)	H_2_SO_4_ (1 M)	24.6 Wh/kg 0.6 kW/kg (1 A/g)	92% (10,000)	[89]
BC–SA * composite	KOH and O doping	302 F/g (5 mV/s)	KOH (6 M)	15.6 Wh/kg 20 kW/kg (20 A/g)	93.8% (10,000)	[79]
Pomelo peel	N/P co-doping by NH_4_H_2_PO_4_	314 F/g (5 mV/s)	Li_2_SO_4_ (2 M)	36 Wh/kg 1000 W/kg (1 A/g)	99% (10,000)	[90]
Tea leaves	NaOH activation Ni(OH)_2_ decoration	945 F/g (10 mV/s)	Na_2_SO_4_ (1 M)	58 Wh/kg 6.32 kW/kg (1 A/g)	>94% (10,000)	[91]
Rice straw	KOH activation N/O doping	324 F/g (2 mV/s)	EMI-TFSI (N.A.)	48.9 Wh/kg 750 W/kg (0.5 A/g)	95% (10,000)	[92]
Cladophora glomerata	KOH/H_2_SO_4_/HNO_3_/FeCl_3_	368 F/g (5 mV/s)	KCl (3 M)	41.5 Wh/kg 900 W/kg (1 A/g)	91.3% (10,000)	[72]
Kitchen waste	Molten K_2_CO_3_ method	237.4 F/g (10 mV/s)	Na_2_SO_4_ (1 M)	4.2 Wh/kg 8 kW/kg (0.5 A/g)	95% (10,000)	[93]
Human hair	KOH activation PPy	358 F/g (5 mV/s)	H_2_SO_4_ (1 M)	53.3 Wh/kg 408.5 W/kg (0.5 A/g)	92.7% (10,000)	[94]
Celery	KOH/N doping/PANI decoration	402 F/g (5 mV/s)	H_2_SO_4_ (1 M)	178.2 Wh/kg 473.3 W/kg (1 A/g)	97% (10,000)	[95]
*Flammulina velutipes*	MgO decoration N and O doping	470.5 F/g (200 mV/s)	Na_2_SO_4_ (1 M)	26.1 Wh/kg 1.0 kW/kg (0.5 A/g)	100% (10,000)	[96]
Coffee grounds	KOH activation Erbium-doped graphene quantum dot decoration	699 F/g (5 mV/s)	KOH (2 M)	94.5 Wh/kg 1.3 kW/kg (1 A/g)	81% (5000)	[97]

* Capacitance data were collected from the three-electrode system of electrode material, while the electrolyte, energy/power densities, and stability are related to the fabricated SC devices. For simply the distinguishing of energy density and power density, data of power density was marked with underline.

## Data Availability

Data sharing is not applicable to this article.
